# A stable implementation measure of multi-transmitting formula in the numerical simulation of wave motion

**DOI:** 10.1371/journal.pone.0243979

**Published:** 2020-12-15

**Authors:** Jie Su, Zhenghua Zhou, Yuandong Li, Bing Hao, Qing Dong, Xiaojun Li

**Affiliations:** 1 College of Transportation Science & Engineering, Nanjing Tech University, Nanjing, China; 2 Faculty of Urban Construction, Beijing University of Technology, Beijing, China; Mirpur University of Science and Technology, PAKISTAN

## Abstract

The Multi-Transmitting Formula (MTF) proposed by Liao et al. is a local artificial boundary condition widely used in numerical simulations of wave propagation in an infinite medium, while the drift instability is usually caused in its numerical implementation. In view of a physical interpretation of the Gustafsson, Kreiss and Sundström criterion on numerical solutions of initial-boundary value problems in the hyperbolic partial differential equations, the mechanism of the drift instability of MTF was discussed, and a simple measure for eliminating the drift instability was proposed by introducing a modified operator into the MTF. Based on the theory of spherical wave propagation and damping effect of medium, the physical implication on modified operator was interpreted. And the effect of the modified operator on the reflection coefficient of MTF was discussed. Finally, the validity of the proposed stable implementation measure was verified by numerical tests of wave source problem and scattering problem.

## Introduction

For the numerical simulations of near-field wave motions and the response of geological structures, the control equations of different media should be determined to obtain the reliable wave propagation characteristics [1–5]. Moreover, we need to truncate models of media in a finite-computational domain by introducing artificial boundaries. Inappropriately set artificial boundary conditions might incur spurious reflections, which not only affect the computational precision at inner grid nodes and boundary nodes and the resolution of wave-field simulation, but also interfere with the response of geological structure [6–8]. Numerous studies have been conducted on artificial boundary conditions since the late 1960s [6, 9–19]. Specifically, the Multi-Transmitting Formula (MTF), which is a local artificial boundary condition proposed by Liao, et al. [20, 21], has been favored because of its simple physical concept, wide adaptability, as well as easy implementation of decoupled high-precision numerical simulations of wave motions and for the wave scattering problem, its unique advantages are more obvious [20, 22–26].

Similar to other local artificial boundary conditions, the MTF is subjected to numerical instability problems in implementation of MTF into the numerical simulation by time-step integration, such as the high-frequency oscillation instability and drift instability. In regards to the high-frequency instability, its mechanism has been clarified, and measures that attempt to eliminate this instability have been proposed [27–30]. For drift instability, the mechanism of high-order drift instability has been explored by using numerical experiments, and a measure has been suggested to suppress it [31]. By reducing the order of MTF to order 1, the drift instability can be suppressed to a certain extent. However, the precision of MTF-1 is not enough to meet the needs of engineering application. Especially, a small computational domain which brings a relatively large incident angle between artificial boundary and input will amplify the drift instability. Therefore, it is worthwhile to further elaborate on the mechanism of drifting instability and to propose corresponding elimination measures. The numerical stability of local artificial boundary conditions is mathematically equivalent to that of the initial-boundary value problems of the hyperbolic partial differential equations. Moreover, for the latter the necessary and sufficient condition for one-dimensional numerical stability, known as the Gustafsson, Kreiss and Sundström (GKS) criterion [32], has been identified. To increase the applicability of this criterion to multi-dimensional case and simplify this complex mathematical theorem, the existing study interprets physical implication of this criterion, i.e., the internal traveling waves which satisfy both artificial boundary conditions and internal motion equations are not allowed [32–34].

In this study, based on above interpretation and combined with decoupled numerical simulations [21, 35], the mechanism of drift instability was clarified, and a simple measure for eliminating drift instability in the numerical simulation of wave motions was proposed, which is to introduce a modified operator $\gamma B_{0}^{0}$ into the MTF. Moreover, the physical implication of $\gamma B_{0}^{0}$ was explained by the spherical wave propagation principle and the medium damping effect; then, the effect of the $\gamma B_{0}^{0}$ on numerical simulation accuracy was analyzed. Finally, the validity of the proposed stability measure was verified by numerical experiments of wave source problem and scattering problem.

## Mechanism of and eliminating measure for MTF drift instability

**Fig 1:**
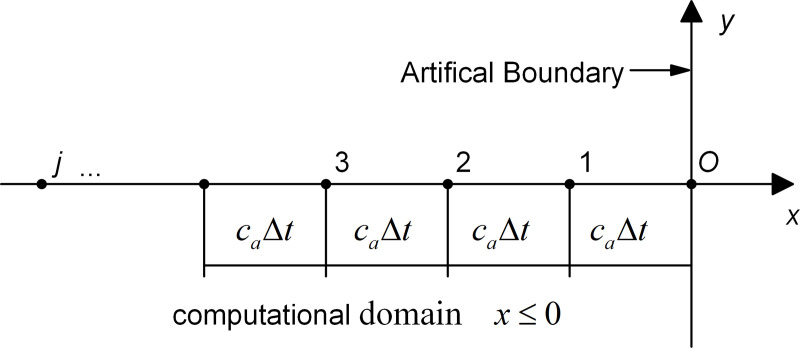
Schematic diagram of the artificial boundary node *o* and MTF computational points.

The *x*-axis is set to be the outer normal of the artificial boundary, and the origin, *o*, coincides with a boundary node, as shown in Fig 1. The displacement of a one-way wave motion propagating forward along the *x*-axis, denoted as *u*(*t*,*x*), is a function of time *t* and coordinate *x*.

Based on the simulation of wave motion, the MTF can be expressed as [24]:

$$u_{0}^{p+1}=\sum_{j=1}^{N}{\left(-1\right)}^{j+1}C_{j}^{N}u_{j}^{p+1-j}$$ (1)

where *N* is the MTF order, $u_{j}^{p}=u\left(p\Delta t,-jc_{a}\Delta t\right)$, *p* and *j* are arbitrary integers, Δ*t* is the time step, *c*_*a*_ is the artificial wave velocity, and $C_{j}^{N}=N!/[\left(N-j\right)!j!]$.

The backward moving operator $B_{m}^{n}$ is defined as [24]:

$$B_{m}^{n}u_{j}^{p}=u_{j+m}^{p-n}$$ (2)

The operator satisfies the following operation rules:

$$B_{m}^{n}B_{s}^{r}=B_{m+s}^{n+r}$$ (3)

Using the backward moving operator, the MTF can be expressed as:

$${\left(B_{0}^{0}-B_{1}^{1}\right)}^{N}u_{0}^{p+1}=0$$ (4)

where $B_{0}^{0}$ is the unit operator.

In the discrete model, the harmonic form of the interior node solution that satisfies the numerical integral stability condition of the nodal motion equation in the computational domain of x < 0 can be expressed as [33]:

$$u_{j}^{p}={\left(\exp\left(i\omega \Delta t\right)\right)}^{p}{\left(\exp\left(ik\Delta x\right)\right)}^{-j}$$ (5)

where *ω* and *k* are the circular frequency of the steady-state one-way wave and the apparent wavenumber along the x-axis, respectively, and they are real numbers with the same signs.

The numerical stability of local artificial boundary conditions is mathematically equivalent to that of the initial-boundary value problems of the hyperbolic partial differential equations. The necessary and sufficient condition for one-dimensional numerical stability of the latter is known as the GKS criterion. According to the GKS criterion, in order to achieve the stability of MTF against any wave motions and prevent internal traveling waves from entering the computational domain, the MTF boundary conditions (Eq (4) should be not satisfied for the internal traveling wave solution (Eq (5)) [33, 34]. Substituting Eq (5) into Eq (4) and combining it with moving operator $B_{m}^{n}$, the stability requirement of MTF for numerical integration can be expressed as:

$${\left[1-\exp\left(-i\left(\omega \Delta t+k\Delta x\right)\right)\right]}^{N}\neq 0$$ (6)

Because Δ*t* and Δ*x* are positive real numbers, *ω* and *k* are real numbers with the same signs, the condition (Eq (6)) holds for all non-zero wavenumbers and non-zero frequencies, with the exception of zero frequency and zero wavenumber. In the case of zero frequency and zero wavenumber, the components of the numerical solution enter the computational domain through the boundary, which caused the violation of GKS criterion and leads to the drift instability. Based on this explanation, we proposed to introduce a small positive parameter into the boundary conditions in the numerical implementation of MTF, and Eq (4) should be rewritten as:

$${\left[\left(1+\gamma \right)B_{0}^{0}-B_{1}^{1}\right]}^{N}u_{0}^{p+1}=0$$ (7)

where *γ* is a small positive value close to 0. It is easy to understand that this measure of numerical implementation in the MTF guarantees that the GKS criterion is met in the case with a zero frequency and zero wavenumbers. At the same time, the small value of *γ* incurs only a slight effect on the computational precision of the numerical simulations over the entire computational domain.

In the next section, the spherical wave propagation principle and the medium damping effect were utilized to analyze the physical implication of the modified operator, $\gamma B_{0}^{0}$.

## Physical implication of $\gamma B_{0}^{0}$

**Fig 2:**
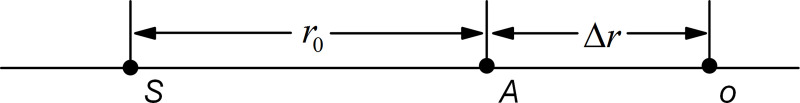
Schematic diagram of the source and observation points.

The spherical wave generated by the point source *S* is assumed to propagate outward at a wave velocity *c* (as shown in Fig 2). The distance between the observation point *A* and point source *S* is *r*_0_, and the distance between the observation point *o* and point source *S* is *r*_0_+Δ*r*. Assuming the displacement of point *A* is known to be *u*_*A*_(*t*), the displacement of point *o* can be obtained according to the spherical wave propagation principle:

$$u_{o}\left(t\right)=\alpha u_{A}\left(t-\Delta r/c\right)$$ (8)

where *α* is the medium geometry diffusion factor, and *α* = *r*_0_/(*r*_0_+Δ*r*) = 1/(1+Δ*r*/*r*_0_). Assuming $\bar{\gamma }=\Delta r/r$, then we had $\alpha =1/\left(1+\bar{\gamma }\right)$. According to operation rules of the moving operator $B_{m}^{n}$, Eq (7) can be expanded as:

$$u_{0}^{p+1}=\sum_{j=1}^{N}{\left(-1\right)}^{j+1}C_{j}^{N}\frac{u_{j}^{p+1-j}}{{\left(1+\gamma \right)}^{j}}$$ (9)

It is known through comparison with the MTF that according to the MTF with $\gamma B_{0}^{0}$, the displacements of the inner nodes should be multiplied by the corresponding factor when extrapolating the displacement of the artificial boundary nodes with the displacements of the inner nodes.

For the sake of simplicity but without a loss of generality, let the MTF order *N* = 2 in Eq (9), and thus:

$$u_{0}^{p+1}=\frac{2}{1+\gamma }u_{1}^{p}-\frac{1}{{\left(1+\gamma \right)}^{2}}u_{2}^{p-1}$$ (10)

Eq (10) can be rewritten as:

$$u_{0}^{p+1}=\frac{1}{1+\gamma }u_{1}^{p}+\frac{1}{1+\gamma }\left(u_{1}^{p}-\frac{1}{1+\gamma }u_{2}^{p-1}\right)$$ (11)

After simplification, Eq (11) can also be expressed as:

$$u_{0}^{p+1}={\tilde{u}}_{1}^{p}+\Delta {\tilde{u}}_{0}^{p+1}$$ (12)

where:

$${\tilde{u}}_{1}^{p}=\frac{1}{1+\gamma }u_{1}^{p},\;\Delta {\tilde{u}}_{0}^{p+1}=\frac{1}{1+\gamma }\Delta {\tilde{u}}_{1}^{p}$$

and

$$\Delta {\tilde{u}}_{1}^{p}=u_{1}^{p}-{\tilde{u}}_{2}^{p-1},\;{\tilde{u}}_{2}^{p-1}=\frac{1}{1+\gamma }u_{2}^{p-1}$$

Eq (12) is another expression of the second-order MTF with $\gamma B_{0}^{0}$, which is equivalent to “Eq (22)” on page 178 of Reference [25]. It can be seen that both the incident wave and the error wave propagate in the form of spherical waves with *α* = 1/1+*γ*. Therefore, it can be concluded that the MTF with $\gamma B_{0}^{0}$ considers the geometric diffusion characteristics of the medium in the numerical simulation of wave motion.

In the following, the second-order MTF is still used as an example to provide another physical explanation of the MTF with $\gamma B_{0}^{0}$. For the condition of *N* = 2, we can obtain [6]:

$$u_{0}^{p+1}=2u_{1}^{p}-u_{2}^{p-1}$$ (13)

assuming

$${\tilde{u}}_{j}^{p}=\exp\left(-\mu p\right)u_{j}^{p}$$ (14)

where exp(−*μp*) is the introduced damping factor. According to the assumption in Eq (14), Eq (13) can be rewritten as:

$${\tilde{u}}_{0}^{p+1}=2\exp\left(-\mu \right){\tilde{u}}_{1}^{p}-\exp\left(-2\mu \right){\tilde{u}}_{2}^{p-1}$$ (15)

For the condition *μ* = ln(1+*γ*), we can obtain:

$${\tilde{u}}_{0}^{p+1}=2\frac{1}{1+\gamma }{\tilde{u}}_{1}^{p}-\frac{1}{{\left(1+\gamma \right)}^{2}}{\tilde{u}}_{2}^{p-1}$$ (16)

Through comparison of Eqs (16) and (10), it is easy to recognize that they are equivalent. Accordingly, it is explained that the MTF with $\gamma B_{0}^{0}$ also introduces damping characteristics of the medium in the numerical simulation of wave motion.

Based on the two explanations of physical of the MTF with $\gamma B_{0}^{0}$ described above, the MTF with $\gamma B_{0}^{0}$ either consider the geometric diffusion characteristics of the medium or introduce the damping characteristics of the medium, thereby the two explanations of physical implications indicate that the MTF with $\gamma B_{0}^{0}$ introduces the absorption mechanism of the medium to wave motion. Moreover, inspired by the introduction of the damping characteristics of the medium, it is worthwhile to explore how much damping that is proportional to the velocity of the particle motion should be introduced to eliminate the drift instability of the MTF, which is an issue that will be studied in the future.

## Accuracy analysis of modified operator $\gamma B_{0}^{0}$

The accuracy of the artificial boundary is usually expressed by the reflection coefficient. In the steady-state case, the reflection coefficient of the artificial boundary is generally defined as [24, 25]:

$$R=\left|\frac{U_{0}^{R}}{U_{0}^{I}}\right|$$ (17)

where $U_{0}^{I}$ and $U_{0}^{R}$ are the amplitudes of the incident plane harmonic wave and the reflected plane harmonic wave, respectively, at the boundary node. If the total displacement of the boundary node, *U*_0_, is expressed as $U_{0}=U_{0}^{I}+U_{0}^{R}$, we can obtain:

$$R=\left|\frac{U_{0}}{U_{0}^{I}}-1\right|$$ (18)

Next, the reflection coefficients of the MTF with $\gamma B_{0}^{0}$ are discussed in two limit cases, Case *A* and Case *B*, in which the total wave field is composed only of incident waves in Case *A* and total wave field is composed of both incident waves and fully developed reflected waves in Case *B*.

### Case *A*

For steady-state wave motions, Eq (9) can be expressed as:

$$U_{0}=\sum_{j=1}^{N}{\left(-1\right)}^{j+1}C_{j}^{N}a^{-j}U_{j}/{\left(1+\gamma \right)}^{j}$$ (19)

where *U*_*j*_(*j* = 0,1,2,3,…) denotes the amplitude of motion at the computational point *j* (Fig 1), *a* = exp(*iω*Δ*t*), $i=\sqrt{‐1}$, and *ω* is the circular frequency.

In the calculation of the reflection coefficient, the term related to time appears in expression of the total displacement and the incident displacement at the same time, which can be eliminated finally during the calculation progress. Considering that the time factor has no influence on the result of reflection coefficient in this case, the time factor (exp(*iωp*Δ*t*)) in the discrete form of the incident plane harmonic wave is ignored (here and hereinafter), and the discrete form can be expressed as:

$$U_{j}^{I}=U_{0}^{I}a_{x}^{j}$$ (20)

where $U_{j}^{I}$ and $U_{0}^{I}$ are amplitudes of the incident harmonic wave at the discrete nodes, *x* = *jc*_*a*_Δ*t*, and the artificial boundary node, *x* = 0, respectively. Moreover:

$$a_{x}=\exp\left(i\omega c_{a}\Delta t/c_{x}\right)$$ (21)

where *c*_*x*_ is the apparent wave velocity propagating along the x-axis, and *a*_*x*_ represents the phase change of displacement when the traveling distance of incident plane wave is *c*_*a*_Δ*t* on the x-axis. Assuming the wave velocity of the incident plane wave to be *c* and the incident angle to be *θ*, thus *c*_*x*_ = *c*/cos*θ*. Assuming *c*_*a*_ = *c*, we can obtain:

$$a_{x}=\exp\left(i\omega \Delta t\cos\theta \right)$$ (22)

In this case, $U_{j}=U_{j}^{I}$. According to Eq (20), we can have:

$$U_{j}=U_{0}^{I}a_{x}^{j}$$ (23)

Substituting Eq (23) into Eq (19) results in:

$$U_{0}=b_{I}U_{0}^{I}$$ (24)

where $b_{I}=\sum_{j=1}^{N}{\left(-1\right)}^{j+1}C_{j}^{N}a^{-j}a_{x}^{j}/{\left(1+\gamma \right)}^{j}$. Using the binomial Equation ${\left(1+x\right)}^{N}=1+\sum_{j=1}^{N}C_{j}^{N}x^{j}$, *b*_*I*_ can be expressed as:

$$b_{I}=1-{\left[1-a^{-1}a_{x}/\left(1+\gamma \right)\right]}^{N}$$ (25)

Substitution of Eq (24) into Eq (18) results in:

$$R={\left|1-a^{-1}a_{x}/\left(1+\gamma \right)\right|}^{N}$$ (26)

Substitution of *a* and *a*_*x*_ into Eq (26) leads to:

$$R={\left|1-\frac{\exp\left[i2\pi \frac{\Delta t}{T}\left(\cos\theta -1\right)\right]}{1+\gamma }\right|}^{N}$$ (27)

where period *T* = 2*π*/*ω*.

### Case *B*

For Case *B*, the incidence of a plane scalar wave is discussed here. *c*_*a*_ is assumed to be equal to the scalar wave velocity *c*, and the incident angle is *θ*. Therefore, *c*_*x*_ = *c*/cos*θ*. Because the total displacement at the discrete node, *x* = −*jc*_*a*_Δ*t*, on the x-axis, is composed of the incident wave and the fully developed reflected wave, we can derive:

$$U_{j}=U_{j}^{I}+U_{j}^{R}$$ (28)

where $U_{j}^{I}$ is the incident wave displacement, given by Eq (20), and $U_{j}^{R}$ is the reflected wave displacement, which can be determined according to:

$$U_{j}^{R}=U_{0}^{R}a_{x}^{-j}$$ (29)

Substituting Eqs (28), (20) and (29) into Eq (19) results in:

$$U_{0}=b_{I}U_{0}^{I}+b_{R}U_{0}^{R}$$ (30)

where *b*_*I*_ is determined by Eq (25), and *b*_*R*_ is determined by:

$$b_{R}=1-{\left(1-a^{-1}a_{x}^{-1}/1+\gamma \right)}^{N}$$

Substituting $U_{0}=U_{0}^{I}+U_{0}^{R}$ into Eq (30) results in:

$$\frac{U_{0}^{R}}{U_{0}^{I}}=-\frac{1-b_{I}}{1-b_{R}}$$ (31)

According to the definition of the reflection coefficient, namely Eq (17), substituting *b*_*I*_ and *b*_*R*_ into Eq (31) results in:

$$R={\left|\frac{1-\frac{a^{-1}a_{x}}{1+\gamma }}{1-\frac{a^{-1}a_{x}^{-1}}{1+\gamma }}\right|}^{N}$$ (32)

Substituting *a* and *a*_*x*_ into Eq (32) results in:

$$R={\left|\frac{1-\frac{\exp\left[i2\pi \frac{\Delta t}{T}\left(\cos\theta -1\right)\right]}{1+\gamma }}{1-\frac{\exp\left[-i2\pi \frac{\Delta t}{T}\left(\cos\theta +1\right)\right]}{1+\gamma }}\right|}^{N}$$ (33)

**Fig 3:**
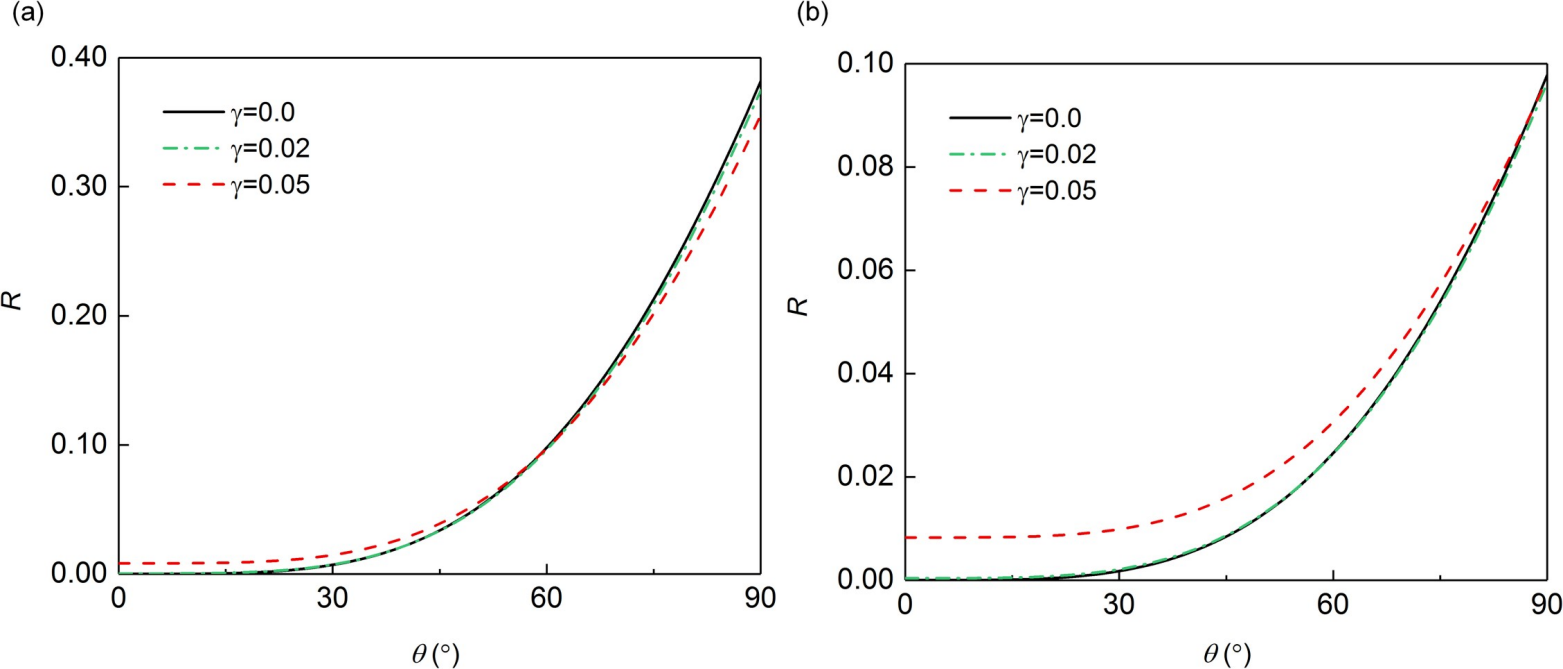
Reflection coefficient in case *A*: (a) **Δ*t*/*T* = 1/10**; (b) **Δ*t*/*T* = 1/20**.

**Fig 4:**
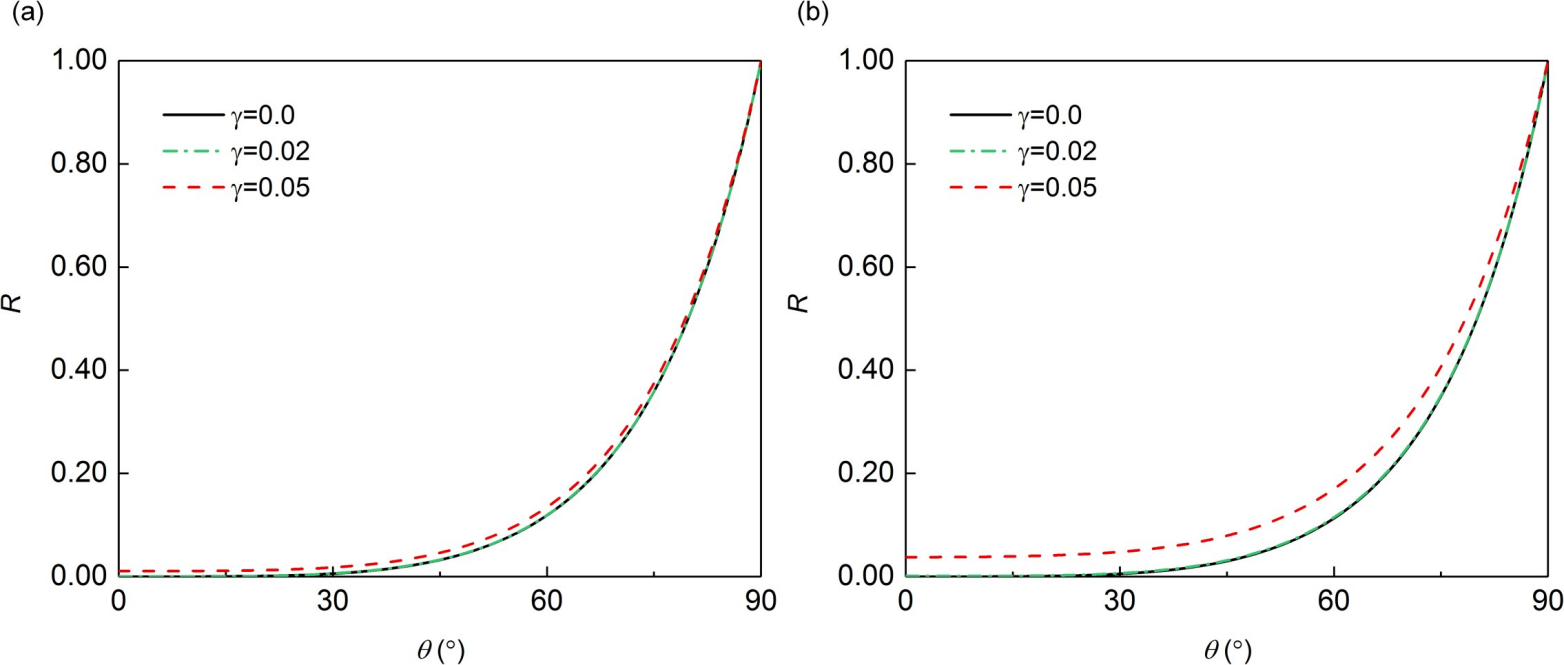
Reflection coefficient in case *B*: (a) **Δ*t*/*T* = 1/10**; (b) **Δ*t*/*T* = 1/20**.

To more intuitively explain the effect of $\gamma B_{0}^{0}$ on the MTF reflection coefficient, we plot the relationship curves between the reflection coefficient and the incident angle corresponding to different *γ*, according to Eqs (27) and (33) for *N* = 2 and Δ*t*/*T* = 1/10 and Δ*t*/*T* = 1/20. As shown in Fig 3, when Δ*t*/*T* was 1/10 or 1/20, the reflection coefficients with the modified operator *γ* of 0.02 became similar to the result that without *γ*, which demonstrated that the modified operator *γ* could have little influence on the computational precision if its value is small. However, with the increase of *γ* to 0.05, the error of the reflection coefficient after adding the *γ* became larger. Moreover, it was seen that when *γ* was 0.05, the overall error of reflection coefficients was larger with the decrease of Δ*t*/*T* from 1/10 to 1/20. As for the case *B* shown in Fig 4, the effect of different value of modified operator on the reflection coefficient is consistent with that shown in case *A* (shown in Fig 3), which indicated that if the added modified operator is small enough, its effect on MTF can be almost ignored.

## Numerical experiments

The decoupling method for the numerical simulation of wave motion [21, 35], which is a combination of the MTF and Lumped mass explicit finite element method, is used to verify the proposed measure of the MTF stably implementation by numerical experiments. Typical examples of the three-dimensional wave source problem and scattering problem are used to examine how this measure effectively eliminates drift instability and ensures the computational precision of numerical simulation.

### Wave source problem

**Fig 5:**
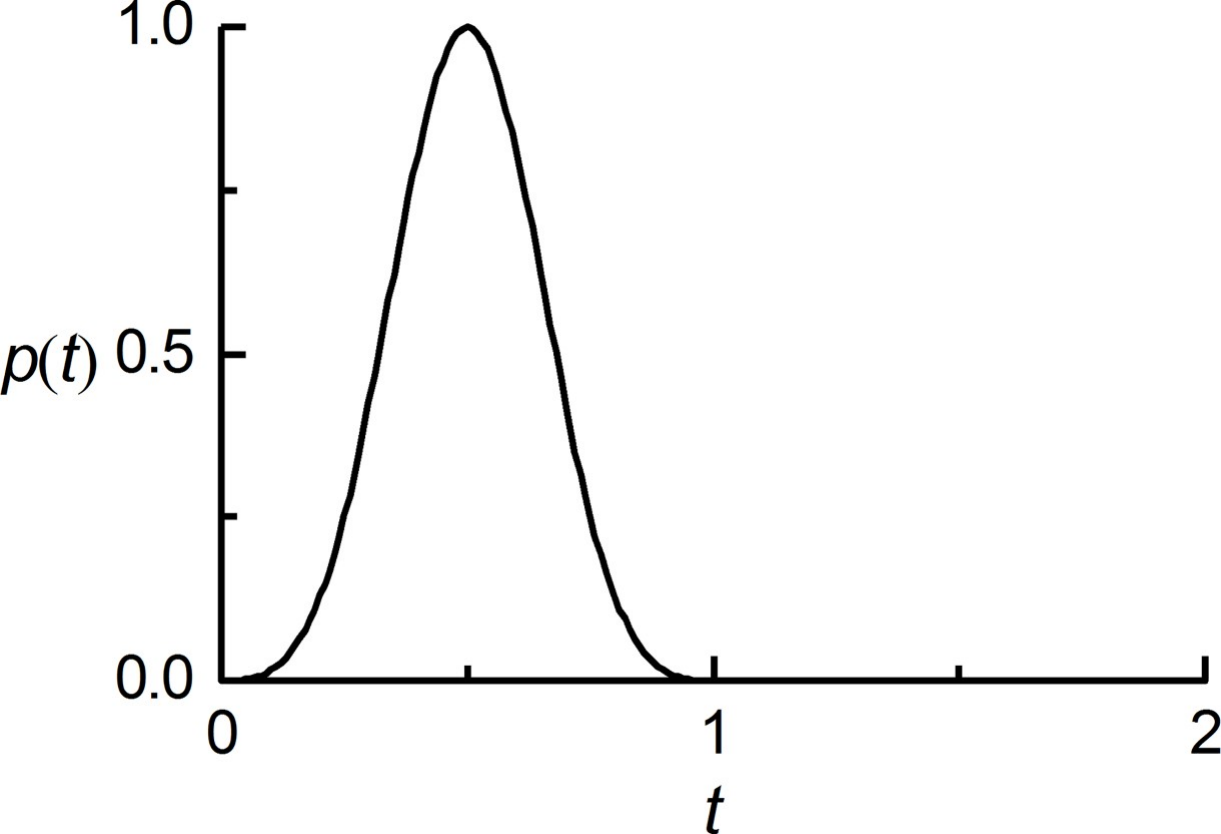
Time function of the concentrated force.

**Fig 6:**
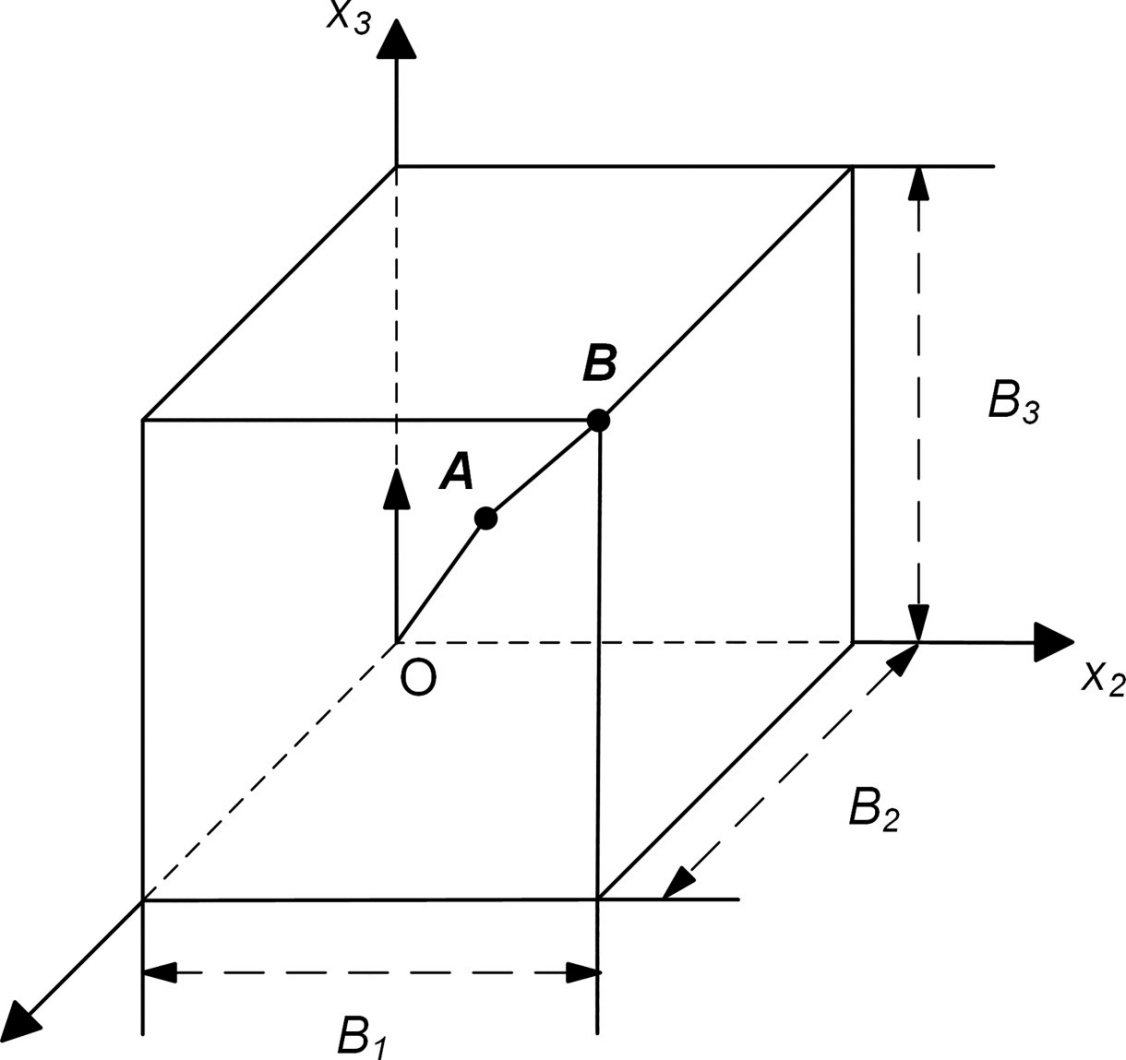
Computational domain in the wave source problem (Quadrant I).

Wave motions generated by a concentrated source of force in a homogeneous, isotropic linear elastic infinite medium are considered. With a Cartesian coordinate system *ox*_1_*x*_2_*x*_3_, the origin of the coordinate system is the same as the action point of the concentrated force. Assumed to act in the direction along the *x*_3_ axis, the concentrated force, *p*(*t*), has a time function of an approximate *δ*-pulse [25] (Fig 5). The numerical simulation of wave motion is performed in the homogeneous box domain, and all of boundaries are the artificial boundary, the sizes of which are represented by *B*_1_, *B*_2_ and *B*_3_ (Fig 6). The computational domain is discretized by the cubic finite elements with a spatial step size of Δ*x*, whose value is determined by the computational precision of the finite element numerical simulation of the wave motion [25], and a system of ordinary differential equations relative to inner nodes can be formed on this basis. The numerical integration of ordinary differential equations is carried out by the central difference method, and the time step Δ*t* of numerical integration is determined by the stability criterion of the central difference method. The discrete equation of nodes on the artificial boundary is a second-order MTF, in which the displacements of the computational points are obtained by the three-point interpolation method with second-order precision based on displacements of finite element nodes [25], the expression is as follows:

$$\begin{array}{l} u_{0}^{p+1}=2u_{1}^{p+1}/\left(1+\gamma \right)-u_{2}^{p-1}/{\left(1+\gamma \right)}^{2}\\ u_{1}^{p}=t_{1,1}{\bar{u}}_{0}^{p}+t_{1,2}{\bar{u}}_{1}^{p}+t_{1,3}{\bar{u}}_{2}^{p}\\ u_{2}^{p-1}=t_{2,1}{\bar{u}}_{0}^{p-1}+t_{2,2}{\bar{u}}_{1}^{p-1}+t_{2,3}{\bar{u}}_{2}^{p-1} \end{array}$$

where

$$t_{1,1}=\left(S-2\right)\left(S-1\right)/2,\;t_{1,2}=S\left(2-S\right),\;t_{1,3}=S\left(S-1\right)/2$$

$$t_{2,1}=\left(2S-1\right)\left(S-1\right),\;t_{2,2}=4S\left(1-S\right),\;t_{2,3}=S\left(2S-1\right)$$

$$S=c_{a}\Delta t/\Delta x$$

in which ${\bar{u}}_{j}^{p}$ and ${\bar{u}}_{j}^{p-1}\left(j=0,1,2\right)$ represent the displacement responses of the finite element nodes at the times of *p*Δ*t* and (*p*−1)Δ*t*, respectively.

**Fig 7:**
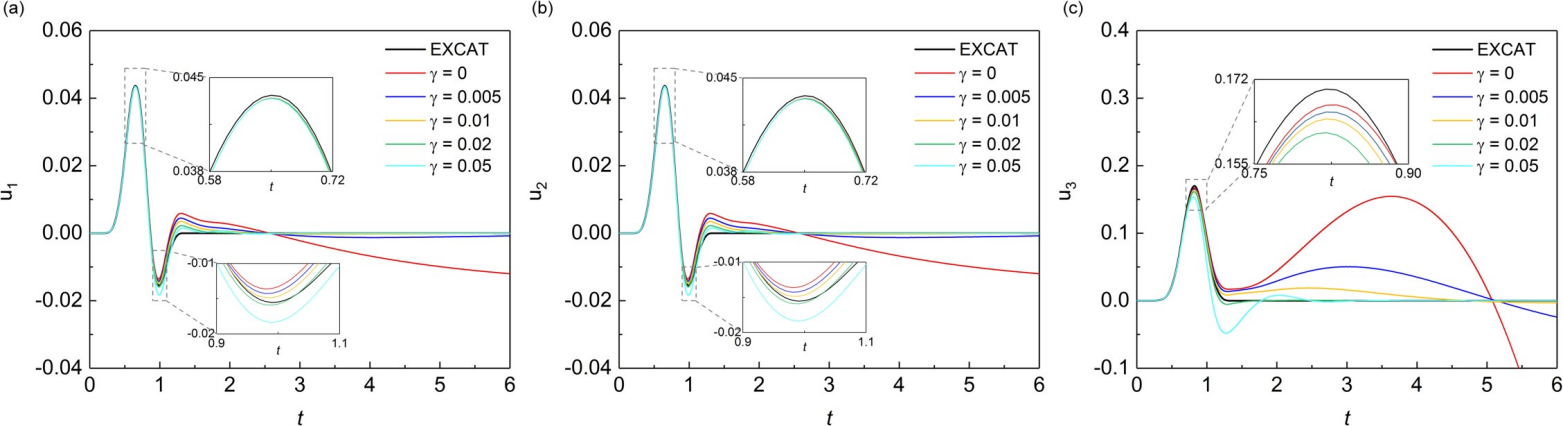
Displacement time-histories of observation point *A*.

**Fig 8:**
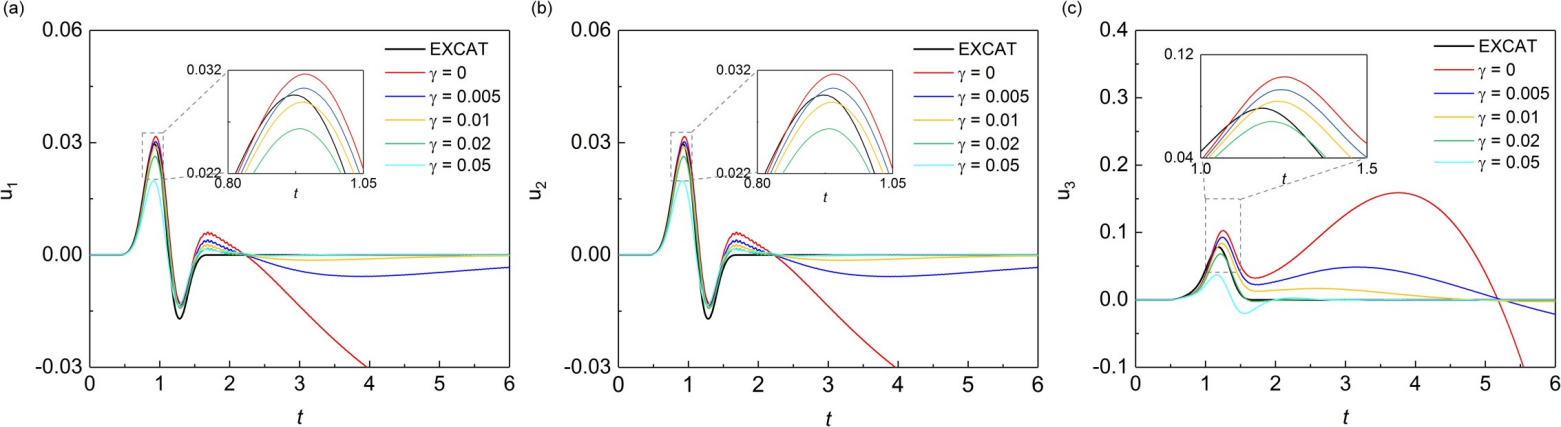
Displacement time-histories of observation point *B*.

The calculation performed in the wave source problem with dimensionless parameters and numerical simulation results of wave motion corresponded to the case of *B*_1_ = *B*_2_ = *B*_3_ = 0.4, medium mass density *ρ* = 1, longitudinal wave velocity $c_{1}=\sqrt{3}$, shear wave velocity *c*_2_ = 1, Δ*x* = 0.02, Δ*t* = 0.01, and artificial wave velocity *c*_*a*_ = 1. The displacements of inner observation point *A* (0.2, 0.2, 0.2) and boundary observation point *B* (0.4, 0.4, 0.4) under different values of *γ* were shown in Figs 7 and 8 and compared with the analytical solution [36] respectively.

As shown in the Fig 7, all three components of displacement responses of the observation point *A* which is inside the computational domain showed significant drift instability when the MTF is without the modified operator. After the addition of the modified operator, the drift instability was effectively suppressed, and as the value of *γ* increased from 0.005 to 0.02, the suppression effect seemed to become better. However, when the value of *γ* continues to increase to 0.05, the error by the addition of modified operator appeared. In the Fig 7C, it can be seen that when the value of *γ* was 0.05, the trough of the component of displacement response in the numerical simulation was obviously deviated from the exact solution, which means that the suppression effect of drift instability became poor at this time. As shown in the Fig 8, it was found that the influence of the value of *γ* on the displacement response of the boundary point B was similar to the influence on the inner point *A*. Besides, the addition *γ* had a worse suppression effect on the component of Z-direction of displacement response, which was due to the fact that the incident angle of the vertical component is larger than that of the horizontal component. In summary, in the wave source problem, a small value of modified operator *γ* can produce a good suppression effect on the drift instability. Whereas, there was an optimal value of *γ*, and the optimal solution of this numerical case seemed to be around 0.02 and less than 0.05.

### Scattering problem

**Fig 9:**
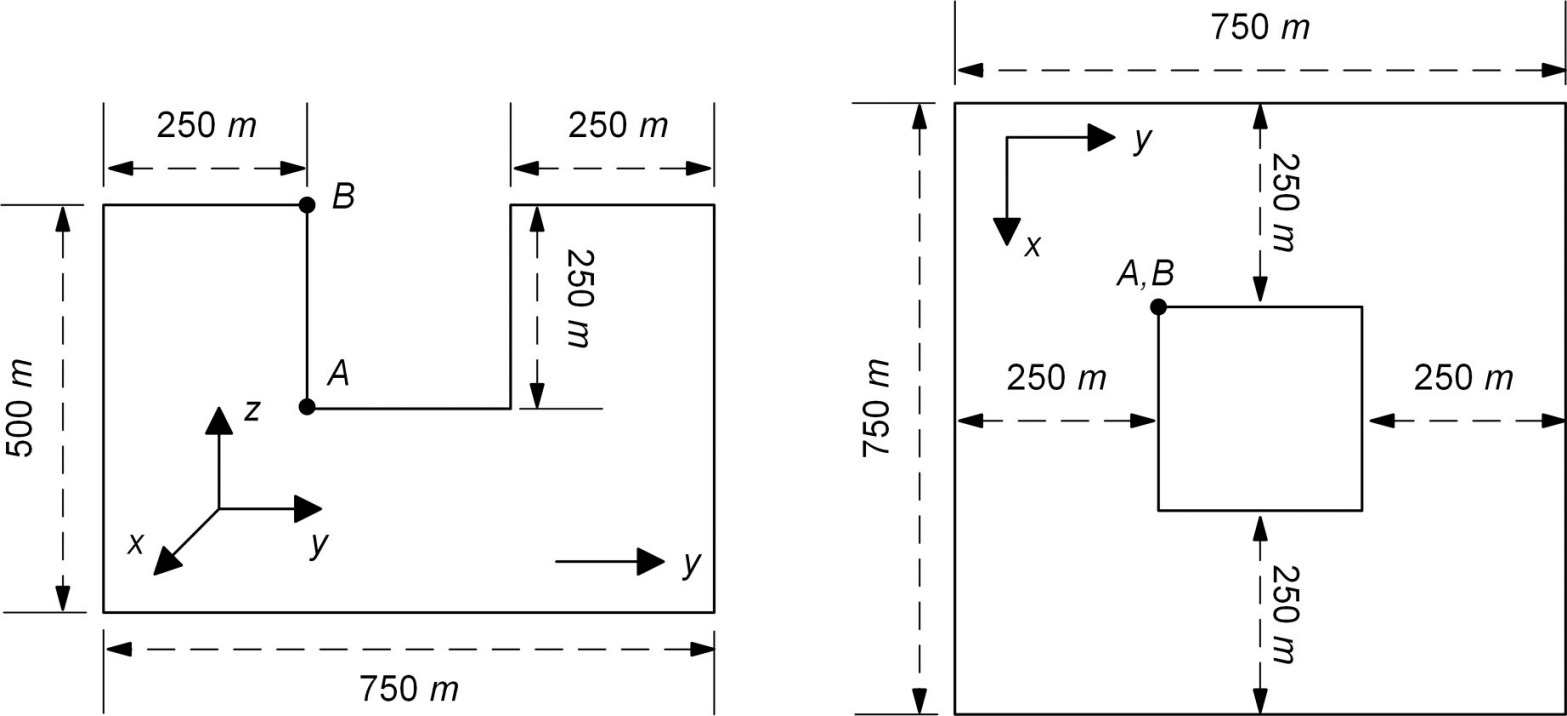
Computational domain in the scattering problem.

The validity of the proposed measure for the elimination of MTF drift instability is examined by numerical simulation of wave motion on the scattering problem. Scattering problem corresponds to scattering field generated by a concave domain (as shown in Fig 9) under vertical incidence plane S-wave, which is located on the free surface of a homogeneous, isotropic semi-infinite elastic space with a medium density *ρ* = 2000 *kg*/*m*^3^, shear wave velocity *c*_*s*_ = 1000 *m*/*s*, and Poisson's ratio *ν* = 0.25. The size of the concave domain is 250 m by 250 m by 250 m, and incidence plane S-wave with vibration direction of particles along the x-axis of the Cartesian coordinate system *oxyz* is an approximate *δ*-pulse (Fig 5) with 1 s pulse width. The numerical simulation of wave motion is carried out in a computational domain of the homogeneous box with top free surface (Fig 9), and both side and bottom boundaries of the box domain are the artificial boundary, and the solution method is the decoupling numerical simulation method used for solving the wave source problem. The computational domain is discretized by cubic finite elements with a spatial step size of Δ*x*, and a system of ordinary differential equations relative to inner nodes can be formed on this basis. The numerical integration of ordinary differential equations is carried out by the central difference method, and the time step Δ*t* of numerical integration is determined by the stability criterion of the central difference method. The discrete equation of nodes on the artificial boundaries is a second-order MTF. In numerical simulation of wave motion, the space step Δ*x* is 12.5 m, the time step Δ*t* is 0.005 s, and the artificial wave velocity *c*_*a*_ is *c*_*s*_.

**Fig 10:**
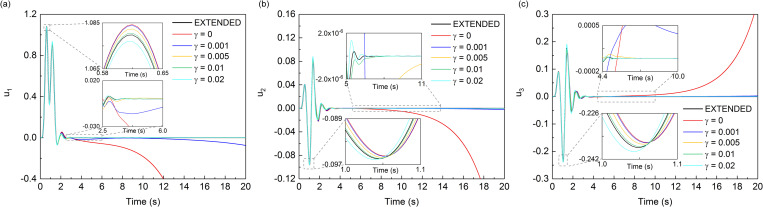
Displacement time-histories of observation point *A*.

**Fig 11:**
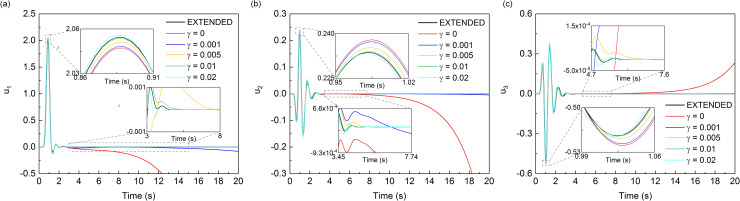
Displacement time-histories of observation point *B*.

Figs 10 and 11 showed the displacement responses at observation points *A* and *B* in Fig 9 respectively, and the comparison with the numerical analytical solution, i.e. artificial boundaries are far enough from the domain of interest so that the numerical solution of this domain is not affected by artificial boundaries during whole analytical time. In the scattering problem, all three components of displacement responses of the observation point *A* and point *B* showed obvious drift instability when the MTF is without the modified operator. As the value of *γ* increased from 0.001 to 0.01, the displacement responses of the observation point *A* and point *B* become closer to the solution of extended computational domain. However, when the values of *γ* exceeded 0.01, it showed a worse suppression effect on the drift instability for observation point *A* and point *B*, especially in the peak of the displacement responses. The variation of the suppression effect with the change of the value of *γ* in scattering problem showed the same trend as that of the wave source problem. But the optimal value of *γ* in the scattering problem seemed to be around 0.01, which was different with that in the source wave problem. From the numerical results of the wave source problem and the scattering problem, it can be seen that the addition of *γ* can effectively suppress the drift instability of MTF, but the value of *γ* should not be too small or too large.

It can be seen from the results of two numerical experiments that the proposed measure is effective in eliminating the drift instability of MTF. The curves in the smaller time windows of Figs 7, 8, 10 and 11 also show that introducing $\gamma B_{0}^{0}$ into MTF has no influence on the computational precision of numerical simulation of wave motion before the drift instability of MTF appears. Theoretically, *γ* can be any small positive number, but it should not be too small when considering the existence of error noise in numerical simulation, and neither should it be too large considering the computational precision of the numerical simulation. It can be seen that the addition of *γ* can effectively suppress the drift instability of MTF, and the range of which is recommend from 0.01 to 0.05 referring to the calculation cases of our work. Though there is no further study on the optimal value of *γ*, the optimal value should be related to the scale of the model computational domain. The general principle is to minimize the value of *γ* under the premise of stable implementation of the MTF in numerical simulations of the wave motions.

## Conclusions

The Mechanism of drift instability in the numerical implementation of MTF is theoretically analyzed, and it reveals that drift instability is caused by the reason that the GKS criterion is violated in the MTF with a zero frequency and zero wavenumber. To eliminate this instability, a simple measure in the numerical simulation of wave motions, introducing a modified operator $\gamma B_{0}^{0}$ into the MTF, was proposed. Due to introduction of the modified operator $\gamma B_{0}^{0}$, the absorption mechanism of the medium to the wave in the numerical simulation of wave motion is drawn into the MTF. In addition, through the accuracy analysis based on the calculation of the reflection coefficient and the verification of numerical experiments, the proposed measure is effective in eliminating drift instability of MTF and has slight influence on the accuracy of numerical simulation under the appropriate small value of *γ*. It is recommended that the value of *γ* should not be too large or too small, the optimal value varies according to the different calculation models. Based on the numerical simulations we have carried, the trial range of value of is suggested to be in 0.01 to 0.05. Besides, our strategy for eliminating the drift instability of MTF is also suitable for all versions of MTF.

## Supporting information

S1 File Data in Figs 3 and 4. (XLSX)

S2 File Data in Figs 7 and 8. (XLSX)

S3 File Data in Figs 10 and 11. (XLSX)

## References

[1] Kim SH, Kim KJ, Blouin SE. Analysis of Wave Propagation in Saturated Porous Media. I. Theoretical Solution. Comput Meth Appl Mech Eng. 2002; 191(37): 4061–4073.

[2] Wei C, Kanthasamy KM. A Continuum Theory of Porous Media Saturated by Multiple Immiscible Fluids: I. Linear Poroelasticity. Int J Eng Sci. 2002; 40(16): 1807–1833.

[3] Heider Y, Markert B, Ehlers W. Dynamic wave propagation in infinite saturated porous media half spaces. Comput Mech. 2012; 49(3): 319–336.

[4] Ba J, Xu W, Fu L, Carcione JM, Zhang L. Rock anelasticity due to patchy-saturation and fabric heterogeneity: A double double-porosity model of wave propagation. J Geophys Res-Solid Earth. 2017; 122(3): 1949–1976.

[5] Ma R, Ba J. Coda and intrinsic attenuations from ultrasonic measurements in tight siltstones. J Geophys Res-Solid Earth. 2020; 125: e2019JB018825.

[6] Liao ZP. Numerical simulation of near-field wave motion. Adv Mech. 1997; 27(2): 193–216.

[7] Zhang Z, Wang G, Harris JM. Multi-component wavefield simulation in viscous extensively dilatancy anisotropic media. Phys Earth Planet Inter. 1999; 114(1): 25–38.

[8] Lan H, Zhang Z. Three-Dimensional Wave-Field Simulation in Heterogeneous Transversely Isotropic Medium with Irregular Free Surface. Bull Seismol Soc Amer. 2011; 101(3): 1354–1370.

[9] Cerjan C, Kosloff D, Kosloff R, Reshef M. A nonreflecting boundary condition for discrete acoustic and elastic wave equations. Geophysics. 1985; 50(4): 705–708.

[10] Sochacki J, Kubichek R, George J, Fletcher WR, Smithson SB. Absorbing boundary conditions and surface waves. Geophysics. 1987; 52(1): 60–71.

[11] Berenger JP. A perfectly matched layer for the absorption of electromagnetics waves. J Comput Phys. 1994; 114(2): 185–200.

[12] Ehrhardt M. Absorbing boundary conditions for hyperbolic systems. Numer Math-Theory Methods Appl. 2010; 3: 1–44.

[13] Sun ZZ, Wu X, Zhang J, Wang D. A linearized difference scheme for semilinear parabolic equations with nonlinear absorbing boundary conditions. Appl Math Comput. 2012; 218(9): 5187–5201.

[14] Lian XM, Shan LY, Sui ZQ. An overview of research on perfectly matched layers absorbing boundary condition of seismic forward numerical simulation. Progress in Geophysics. 2015; 30(4): 1725–1733.

[15] Xu CS, Song J, Du XL, Zhao M. A local artificial-boundary condition for simulating transient wave radiation in fluid-saturated porous media of infinite domains. Int J Numer Methods Eng. 2017; 112(6): 529–552.

[16] Yang DH, Liu E, Zhang ZJ, Teng J. Finite-difference modelling in two-dimensional anisotropic media using a flux-corrected transport technique. Geophys J Int. 2002; 148(2): 320–328.

[17] Cao S, Greenhalgh SA. Attenuating boundary conditions for numerical modeling of acoustic wave propagation. Geophysics. 1998; 63(1): 231–243.

[18] Liu Y, Mrinal KS. An Improved Hybrid Absorbing Boundary Condition for Wave Equation Modeling. J Geophys Eng. 2018; 15(6): 2602–2613.

[19] Wang X, Tang S. Analysis of Multi-Transmitting Formula for Absorbing Boundary Conditions. Int J Multiscale Comput Eng. 2010; 8(2): 207–219.

[20] Liao ZP, Wong HL. A transmitting boundary for the numerical simulation of elastic wave propagation. Int J Soil Dyn Earthq Eng. 1984; 3(4): 174–183.

[21] Liao ZP. A finite element method for near-field wave motion in heterogeneous materials. Earthquake Engineering and Engineering Vibration. 1984; 4(2): 1–14.

[22] Liao ZP, Huang KL, Yang BP, Yuan YF. A Transmitting boundary for transient wave. Scientia Sinica (Series A). 1984; 27(6): 556–564.

[23] Wang X, Tang S. Analysis of Multi-Transmitting Formula for Absorbing Boundary Conditions. J Multiscale Computational Engineering, 2010; 8(2): 207–219.

[24] Liao ZP. Extrapolation non-reflecting boundary conditions. Wave Motion. 1996; 24(2): 117–138.

[25] Liao ZP. Introduction to Wave Motion Theories in Engineering. Beijing: Science Press; 1996.

[26] Shi L, Wang P, Cai Y, Cao Z. Multi-transmitting formula for finite element modeling of wave propagation in a saturated poroelastic medium. Soil Dynam Earthq Eng. 2016; 80: 11–24.

[27] Liao ZP, Liu JB. Fundamental problems in finite element simulation of wave motion. Science in China (Series B). 1992a; 8: 874–882.

[28] Liao ZP, Liu JB. Numerical instabilities of a local transmitting boundary. Earthq Eng Struct Dyn. 1992; 21(1): 65–77.

[29] Xie ZN, Liao ZP. A note for the mechanism of high-frequency instability induced by absorbing boundary conditions. Acta Seismologica Sinica. 2008; 30(3): 302–306.

[30] Dong Q, Zhou ZH, Su J, Liu HY, Song JM. The measure against high frequency oscillating instability of multi-transmitting formula. Technology for Earthquake Disaster Prevention. 2018; 13(3): 571–577.

[31] Li XJ, Liao ZP. The drift instability of local transmitting boundary in time domain. Acta Mechanica Sinica. 1996; 28(5): 627–632.

[32] Gustafsson B, Kreiss HO, Sundstrom A. Stability theory of difference approximations for mixed initial boundary value problems. II. Math Comput. 1972; 26(119): 649–686.

[33] Higdon RL. Numerical absorbing boundary conditions for the wave equation. Math Comput. 1987; 49(179): 65–90.

[34] Liao ZP. Dynamic interaction of natural and man-made structures with earth medium. Earthquake Research in China. 1999; 13(3): 367–408

[35] Liao ZP. A decoupling numerical simulation of wave motion. Developments in geotechnical engineering. 1998; 83: 125–140.

[36] Achenbach JD. Wave Propagation in Elastic Solids. Amsterdam; North-Holland: 1973.

